# Double hemispheric Microdialysis study in poor-grade SAH patients

**DOI:** 10.1038/s41598-020-64543-x

**Published:** 2020-05-04

**Authors:** Ramon Torné, Diego Culebras, Gerard Sanchez-Etayo, Sergio García-García, Guido Muñoz, Laura Llull, Sergio Amaro, Christian Heering, Jordi Blasco, Elizabeth Zavala, Joaquim Enseñat

**Affiliations:** 1 Neurological Surgery Department, Hospital Clinic of Barcelona, Barcelona, Spain; 2 Intensive Care Unit, Hospital Clinic of Barcelona, Barcelona, Spain; 3 Neurological Surgery Department, Hospital Sant Joan de Deu, Barcelona, Spain; 4 Neurology Department, Comprehensive Stroke Unit, Hospital Clinic of Barcelona, Barcelona, Spain; 5 Radiology Department, Angioradiology Section, Hospital Clinic of Barcelona, Barcelona, Spain

**Keywords:** Predictive markers, Cerebrovascular disorders, Stroke

## Abstract

Delayed cerebral ischemia (DCI) is a dreadful complication present in 30% of subarachnoid hemorrhage (SAH) patients. DCI prediction and prevention are burdensome in poor grade SAH patients (WFNS 4–5). Therefore, defining an optimal neuromonitoring strategy might be cumbersome. Cerebral microdialysis (CMD) offers near-real-time regional metabolic data of the surrounding brain. However, unilateral neuromonitoring strategies obviate the diffuse repercussions of SAH. To assess the utility, indications and therapeutic implications of bilateral CMD in poor grade SAH patients. Poor grade SAH patients eligible for multimodal neuromonitoring were prospectively collected. Aneurysm location and blood volume were assessed on initial Angio-CT scans. CMD probes were bilaterally implanted and maintained, at least, for 48 hours (h). Ischemic events were defined as a Lactate/Pyruvate ratio >40 and Glucose concentration <0.7 mmol/L. 16 patients were monitored for 1725 h, observing ischemic events during 260 h (15.1%). Simultaneous bilateral ischemic events were rare (5 h, 1.9%). The established threshold of ≥7 ischemic events displayed a specificity and sensitivity for DCI of 96.2% and 83.3%, respectively. Bilateral CMD is a safe and useful strategy to evaluate areas at risk of suffering DCI in SAH patients. Metabolic crises occur bilaterally but rarely simultaneously. Hence, unilateral neuromonitoring strategies underestimate the risk of infarction and the possibility to offset its consequences.

## Introduction

Delayed cerebral ischemia (DCI) is considered one of the main causes of neurological deficits in subarachnoid hemorrhage (SAH) and it may occur in up to 30% of patients. DCI typically occurs between the fourth and tenth day after bleeding but its pathophysiology remains yet unclear. Nevertheless, various pathological phenomena such as oxidative stress, cortical spreading depolarizations, microvascular thrombosis and disbalances in cerebral microcirculation are known to be involved in its development^1,2^. On the other hand, the role of vasospasm in DCI appearance is nowadays controversial. In fact, angiographic vasospasm resolution induced by novel therapies such as clazosentan, does not correlate neither with DCI prevention nor with functional prognosis^3^. This fact may support the absence of a causal relationship between vasospasm and DCI^4,5^.

In poor grade SAH patients, CMD monitoring may be useful for early detection of potentially salvageable brain tissue exposed to ischemic risk. CMD monitoring provides semiquantitative metabolic measurements of the brain tissue independently of the initial insult. CMD patterns of ischemia (Glucose <0.7 mmol/l and Lactate pyruvate ratio (LPR) > 40) have been consistently associated with high specificity and sensitivity to detect DCI^4,6,7^. However, CMD requires a small surgery, its cost is not despicable and the locally confined nature of the obtained measurements limits their validity to interpret the global state of the brain. One of the major challenges when dealing with CMD is to extrapolate the local and timely delayed measurements it provides. Therefore, determining the location of the catheters is not a trivial issue. CMD catheters are normally placed in the frontal lobe, in the watershed area between the vascular territories of the middle and anterior cerebral arteries (MCA and ACA). In SAH patients, the catheter is consensually placed in the hemisphere where the aneurysm or most of the blood are located. Nevertheless, ischemic events in non-monitored areas will probably be disregarded. Furthermore, it might be difficult to determine which hemisphere is at risk in some ACA aneurysms or diffuse bleeding patterns. Ischemia in the non-monitored hemisphere is not rare and it may represent up to one third of secondary infarctions^8,9^.

Using a “one catheter” monitoring protocol means assuming that the hemisphere hosting more blood is the most susceptible and that information from one side might be extrapolated to identify metabolic changes in remote areas. However, regarding SAH, there is a lack of knowledge on the frequency of silent infarcts and the metabolic response of those contralateral areas to the primary insult. Thus, in the present clinical investigation we sought to assess the utility of a bilateral CMD monitoring protocol in the setting of poor grade SAH patients. Our main objective was to detect bilateral metabolic ischemic events in order to identify silent infarcts. This information may contribute to anticipate DCI diagnosis and may also provide us with therapeutic tools to prevent this feared complication.

## Methodology

All methods were carried out in accordance with relevant guidelines and regulations. The methodology of this report follows the recommendations of the Strengthening the Reporting of Observational Studies in Epidemiology (STROBE) guidelines^10^.

The present research was approved by the institutional review board (Comité ético de investigaciones clínicas del Hospital Clínic de Barcelona. CEIC-Reference number HCB/2018/0390). Moreover, in the absence of conscious patients, first grade relatives signed an informed consent authorizing patient’s inclusion in the bilateral CMD neuromonitoring protocol.

### Patients

Poor grade SAH patients (WFNS 4 or 5 grades) eligible for multimodal neuromonitoring were prospectively collected between January 2017 and January 2019. Included patients were those whose neurological status required maintaining them under sedation and ventilation for at least 48 hours. Patients who survived less than 24 h or in whom CMD monitoring lasted less than 48 h were excluded.

### Management

CMD catheters were implanted bilaterally in MCA/ACA watershed territory just after the surgical or endovascular exclusion of the aneurysm. Aneurysm exclusion modality was decided consensually by our neurovascular board. CMD catheters were implanted in the operating room using a mini burr hole located at Kocher’s point. In patients with surgically treated aneurysms, ipsilateral CMD catheter was placed under direct vision through a small corticectomy. A computed tomography (CT) was performed in the 24 hours following CMD catheters placement to confirm their correct location and to rule out early ischemic lesions due to aneurysm exclusion or as a response to SAH primary insult.

### Microdialysis monitoring

Cerebral microdialysis was performed using a 20 kDa catheter (CMA 70; CMA/Microdialysis, Solna, Sweden) with a membrane length of 10 mm. The intraparenchymal probe was implanted at a depth of 2–3 cm and connected to the perfusion pump (CMA 106; CMA/Microdialysis). Perfusion fluid (CMA T [CMA/Microdialysis], consisting of Na+ 147 mmol/L, K+ 4 mmol/L, Cl 156 mmol/L, pH 6, osmolality 290 mosm/kg) was pumped at a flow rate of 0.3 μl/minute. Conventional microdialysis analysis equipment (CMA 600; CMA/Microdialysis) was implemented to record extracellular fluid concentrations of glucose, pyruvate, lactate, glycerol, and glutamate^11^.

Samples were collected hourly. Data recording was stopped if one or both catheters consistently reported errors. Ischemic events were defined as those observations in which Lactate/Piruvate ratio (LPR) was >40 and Glucose concentration was <0.7 mmol/L.

All monitored patients underwent a safety protocol in order to spot any issues related to such monitoring. The safety protocol included: (1) Daily revision of catheter wounds to identify any CSF leak or purulent material; (2) Once the monitoring was over, the catheter tip was routinely sent to the lab for culture; (3) A CT scan was routinely performed after catheter placement in order to rule out hematomas -any blood collection over 5cc- or any other complication related to the catheter insertion.

### Cerebral blood volumetry and aneurysm location

**Figure 1:**
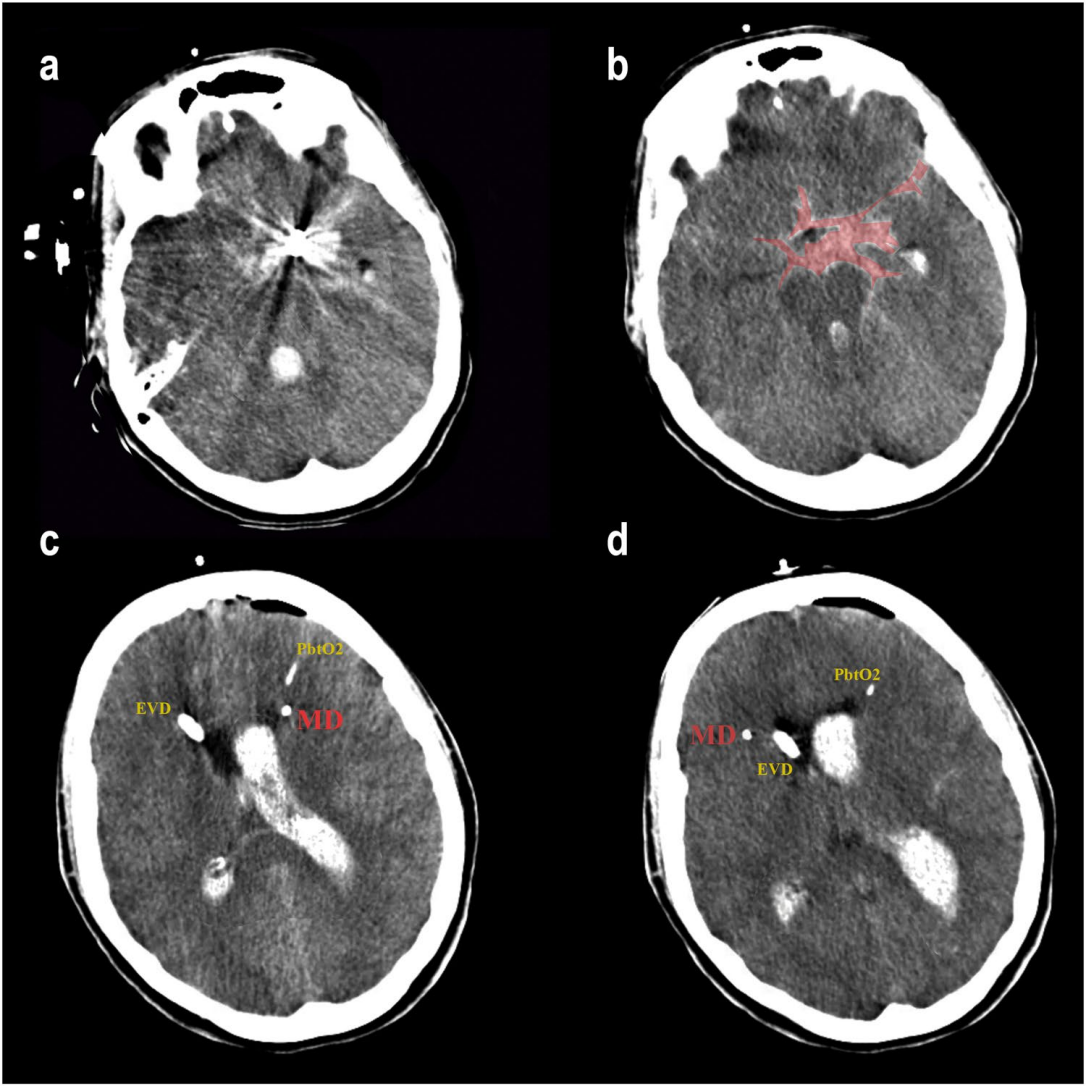
(**a,b**) Computed tomography of a patient with a poor grade aSAH. Left posterior communicating aneurysm (PCom) embolized. More amount of blood in the left basal cistern of the brain. (**c,d**) Bilateral monitorization with two CMD probes. An external ventricular drainage placed on the right side and a PbtO2 probe on the left side. The subarachnoid hemorrhage volume is represented by a red shaded area.

Cerebral blood volumetry in the initial CT was calculated using OSIRIX^12^. Aneurysm’s parent vessel and hemisphere were defined on the AngioCT. The CMD probe located in the hemisphere containing more blood was named “a”, while “b” was reserved for the contralateral one. In those cases, in which differences in volumetry between hemispheres were inferior to 5cc, “a” was used for the catheter ipsilateral to the aneurysm **(**Fig. 1**)**. Anterior communicating artery (AcoA) aneurysms were considered left or right depending on the dominant A1 segment.

### Cerebral magnetic resonance (DCI)

Cerebral magnetic resonance (MRI) was performed prior to patient’s discharge and within 14 days of SAH. This MRI was aimed to find new ischemic lesions not present in the initial CT. Ischemic lesions were classified into two categories: those happening in the brain tissue typically considered at risk and those occurring in silent areas. It was also determined if the ischemic lesions might be related to aneurysm treatment.

### Functional outcome

Functional neurological outcome was assessed at three months after SAH using the modified Rankin scale (mRS). Patients lost during follow up were discarded.

### Statistical analysis

All Statistical analyses were performed using Stata/IC 13.1 for Mac version. Continuous variables were reported as mean (standard deviation) or median (interquartile range). Metabolic records obtained in each hemisphere were compared with Student t-test. Level of significance was established at a *p* level of 0.05 (2-sided). Sensitivity and specificity of ischemic events detected by means of CMD for DCI diagnosis were calculated. In addition, a receiver operating characteristic (ROC) curve analysis was performed to determine the optimal cutoff value of ischemic events for the prediction of DCI.

## Results

Twenty consecutive poor grade aneurysmatic SAH patients admitted to our institution between January 2017 and January 2019 and requiring multimodal monitoring were prospectively recruited for the study. Four patients were excluded: two died within the initial 24 hours and two experienced an accidental removal of the catheters before completing 48 hours of monitoring. The mean patients’ age was 58 years (SD:7.3). Most of the aneurysms were located in the anterior circulation: MCA aneurysm 38% (6/16); ACoA aneurysm 31% (5/16); Pcom aneurysm 13% (2/16); Carotid aneurysm 6% (1/16); AICA aneurysm 6% (1/16) and PICA aneurysm 6% (1/16). All the patients were admitted in poor clinical condition, 56% (9/16) in WFNS 5. The blood assessment of the early CT scan revealed high amount of cisternal and intraventricular blood in most of the patients. The median (IQR) mFisher scale was 4 (0): 88% (14/16) mFisher:4; 6% (1/16) mFisher:3 and 6% (1/16) mFisher:2 **(**Table 1**)**. Mean cisternal blood volume (CBV) was 22cc (SD 13.3) in the hemisphere named “a”, and 12.8cc (SD 7.10) in the contralateral one. In three patients a parenchymal hematoma was additionally present. Differences in cerebral blood volumetry between hemispheres were inferior to 5cc in 50% of the patients.

**Table 1:** Baseline characteristics, grading and clinical outcomes.

Patient	Age	WFNS	mF	IPH	An	SAH Volume (cc)			Ischemic events hours(%)			LPR > 40 in hours (%)			Total (hours)	DCI		Probe Location		mRS (6 m)
						a	b	Total	a	b	B	LPR(a)	LPR(b)	B		(a)	(b)	(a)	(b)	
1	63	4	4	1	MCA	25.9	9.6	35.5	47 (39.5)	7 (5.9)	0	77 (65)	1 (1)	7 (5.8)	119	1	0	PL	N	4
2	72	5	4	0	PICA	26.2	25.8	52	0	1 (1.1)	0	10 (11)	2 (2.1)	0	92	0	0	N	N	6
3	53	5	4	0	ACoA	2.6	2.2	4.8	0	2 (2.8)	0	0	10 (13.9)	0	72	0	0	N	N	2
4	56	5	4	0	ACoA	39.4	32.8	72.2	12 (11.9)	2 (2)	0	58 (57.4)	1 (1)	1 (1)	101	1	0	PL	N	6
5	55	4	2	1	MCA	2.7	1.6	4.3	74 (60)	0	0	78 (62)	0	0	124	1	0	PL	N	1
6	48	4	4	0	ACoA	28.3	27.8	56.1	2 (1.5)	27 (20.5)	1 (1)	3 (2)	62 (47)	29 (23)	132	0	1	N	N	6
7	66	4	3	0	MCA	24.9	2.6	27.5	0	0	0	0	0	0	11	0	0	PL	N	4
8	71	4	4	0	ACoA	24.2	15.3	39.5	2 (1.4)	1 (1)	0	33 (22)	9 (6.2)	22 (15)	145	0	0	N	N	2
9	63	5	4	0	ACI	16.1	19.8	35.9	0	0	0	10 (13)	0	0	77	0	0	N	N	1
10	58	5	4	0	AICA	17.8	10.6	28.4	2 (1.5)	0	0	0	3 (2.2)	0	134	0	0	N	N	4
11	54	5	4	1	MCA	49.8	21	70.8	0	0	0	1 (0.6)	7 (4.1)	0	172	0	0	N	N	6
12	55	5	4	0	MCA	6.1	2.5	8.6	5 (4.8)	1 (1)	0	42 (41)	1 (0.9)	2 (1.9)	103	0	0	PL	N	4
13	51	4	4	0	PCom	23.8	16.3	40.1	1 (1)	19 (11.9)	0	1 (0.6)	80 (0.5)	0	160	0	0	PL	N	3
14	56	5	4	0	ACoA	13.3	14.6	27.9	40 (25)	0	4 (2.5)	41 (35)	0	16 (13.8)	116	1	0	N	N	2
15	50	4	4	0	PCom	14.7	7.7	22.4	0	0	0	0	1 (1)	0	99	0	0	N	N	1
16	51	5	4	0	MCA	20.3	15.2	35.5	0	5 (20.8)	0	1 (4)	5 (21)	0	24	0	0	N	N	2

AcoA: anterior communicating artery; ACoP: posterior communicating artery; AICA: anterior inferior cerebellar artery; An: Aneurysm; B: Bilateral; DCI: Delayed cerebral ischemia; IF: Infarction location; IPH: Intraparenchymal hemorrhage; mF: Modified Fisher score; MCA: middle cerebral artery; mRS: modified rankin scale; N: Normal appearing brain tissue; PICA: Posterior inferior cerebellar artery; PL: Perilesional brain tissue; RLP: lactate/pyruvate ratio; SAH volume: Subarachnoid hemorrhage volume; WFNS: World Federation of Neurosurgical Society.

### CMD monitoring and ischemic events

**Figure 2:**
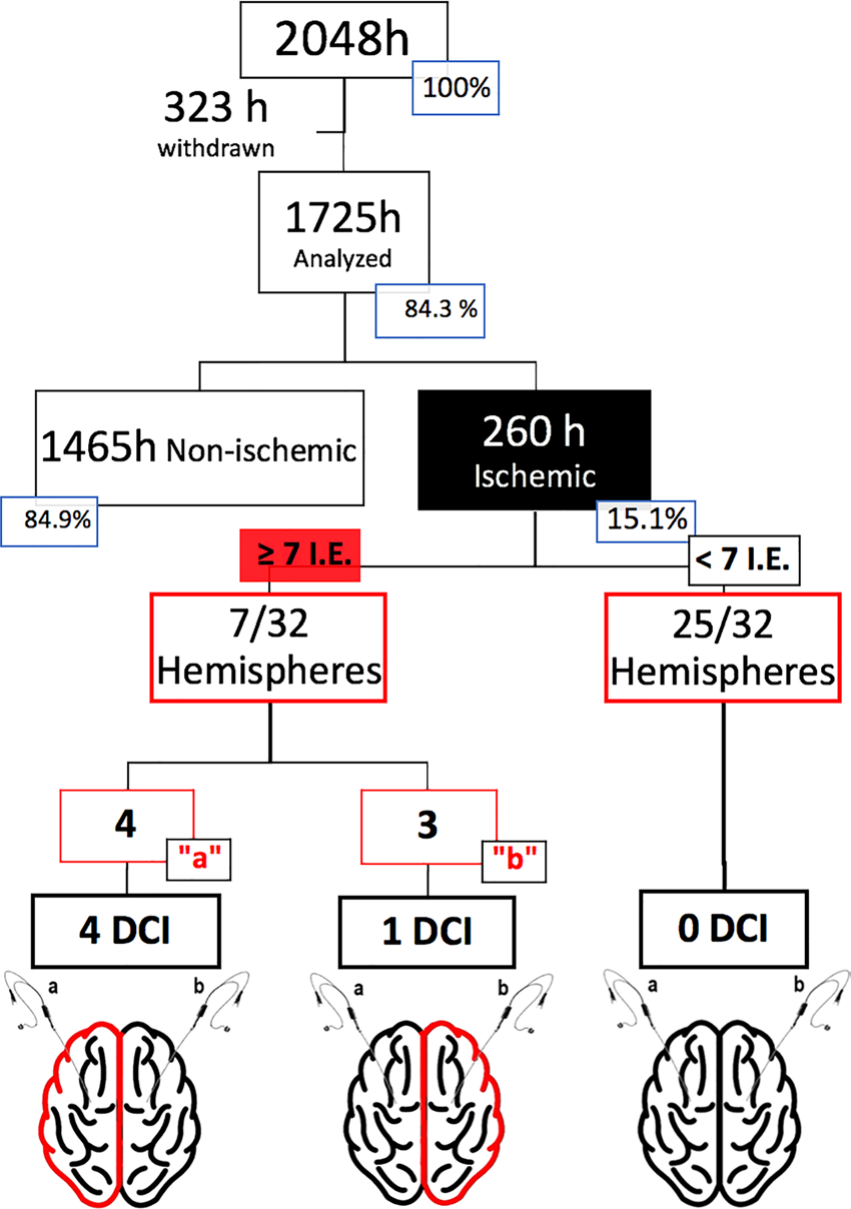
Flow diagram summarizing bilateral hours of monitorization in the study. I.E: Ischemic events; h: total hours of bilateral monitorization; DCI: Delayed cerebral infarction.

Altogether, patients were monitored using bilateral CMD for 2048 hours, consistently during the first week from SAH onset and right after the occlusion of the aneurysm. Mean monitoring time per patient was 108 hours (SD 45.9). A total of 323 hours (16%) were withdrawn from final analysis due to errors in their acquisition or processing. Out of the overall 1725 hours of bilateral observations, 15% revealed an ischemic event in at least one of the probes. Ischemic events located in hemisphere “a” were recorded for a total time of 188 hours (11%; 95% CI, 9.5–12.5). Meanwhile, ischemic events in the silent hemisphere (named “b”), were detected for 72 hours (4%; 95% CI, 3.3–5.2). During 5 hours, ischemic events were simultaneously detected in both catheters **(**Fig. 2**)**.

Ischemic lesions not previously present in the initial CT, were identified in the follow-up MRI of 5 patients, 4 in hemisphere “a” and 1 in hemisphere “b”. All the new infarctions detected were related with delayed ischemia and not attributed to the aneurysm repair procedure. Only 50% (8/16) of the patients had achieved a good functional (mRS 0–3) recovery at the three months follow-up. The median (IQR) mRS at 3 months was 3.5 (2–5). None of the patients initially included were lost at 3 months follow up.

**Figure 3:**
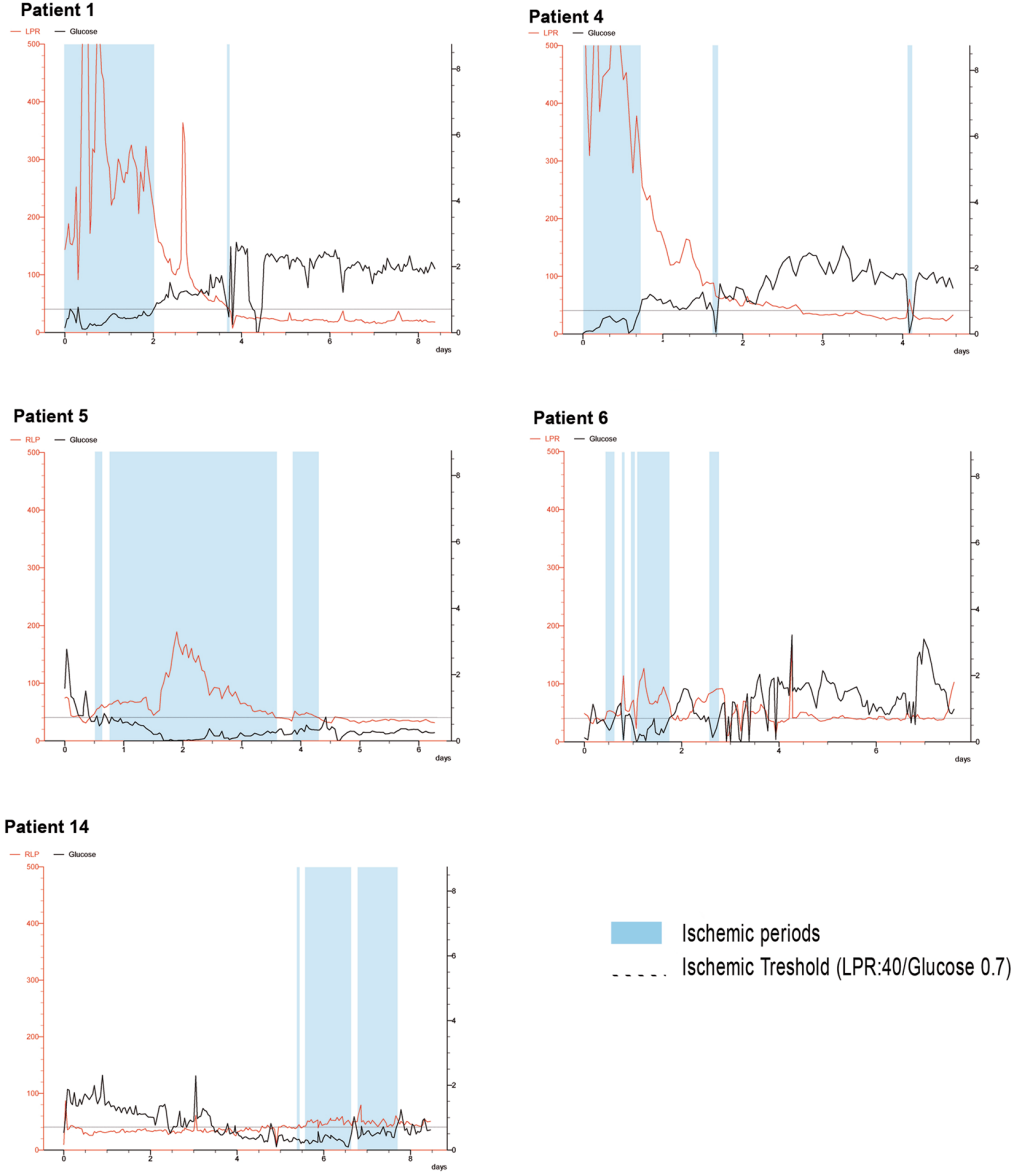
Timeline recordings for patients 1, 4, 5, 6 and 14. The periods in which metabolic ischemic events were registered are represented by a blue shade.

**Figure 4:**
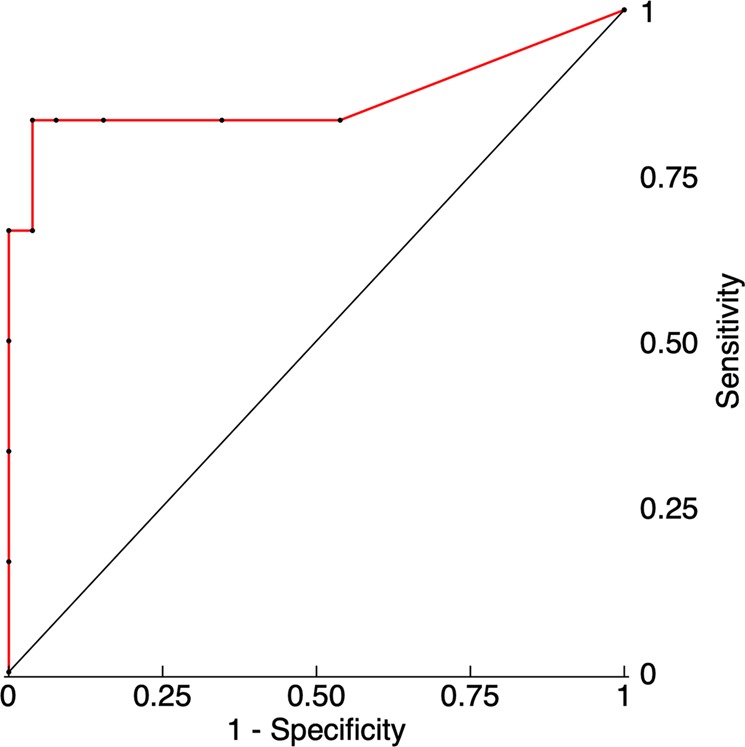
ROC and AUC analyses for bilateral monitorization and DCI prediction.

**Figure 5:**
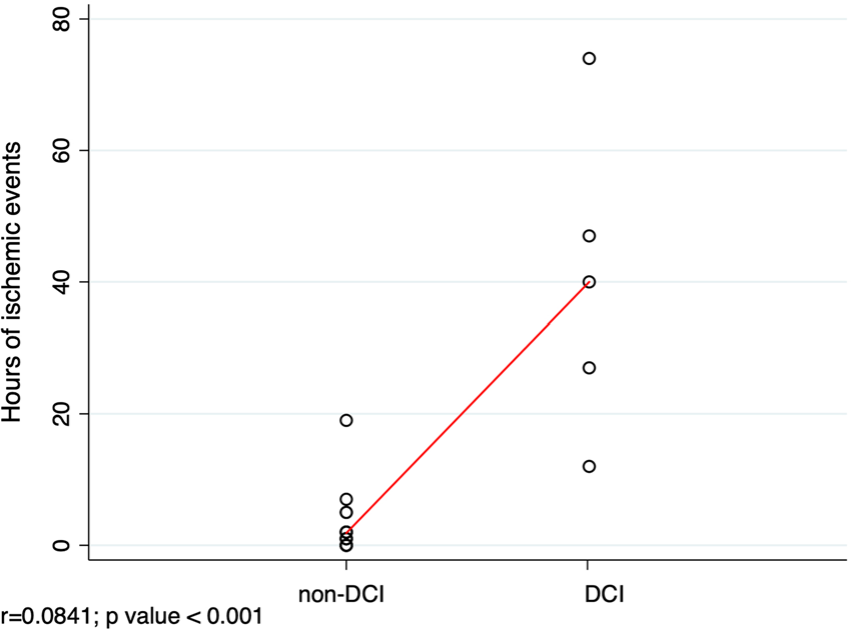
Correlation between hours of ischemic events and MRI infarctions (DCI). Pearson’s bivariate correlation coefficient (r).

Table 1 summarizes the metabolic record of every patient, including ischemic events (LPR > 40) **(**Fig. 3**)**. With a threshold of 7 or more ischemic events, bilateral CMD monitoring strategy was able to predict the occurrence of delayed cerebral infarctions with high specificity (96%) and sensitivity (83%) (AUC: 0.87; 95% CI: 0.61–1) **(**Fig. 4**)**. Figure 5 shows the positive correlation observed between the hours of ischemic events and DCI (r = 0.841, p < 0.001). CMD samples obtained from hemispheres with incident ischemic brain lesions revealed an increase in the mean concentration of every metabolite but glucose when compared to unaffected hemispheres (Table 2).

**Table 2:** Average biochemical parameters observed in the sample, and breakdown by DCI hemispheres and No-DCI hemispheres.

Parameter	All	No DCI Hemisphere	DCI Hemisphere	*P* Value
Glucose (mmol/L)	1.62 (1.58–1.66)	1.79 (1.74–1.83)	0.87 (0.82–0.92)	<0.001
Lactate (mmol/L)	3.94 (3.85–4.03)	3.42 (3.34–3.50)	6.29 (5.99–6.59)	<0.001
Pyruvate (μmol/L)	115.67 (113.57–117)	113.187 (111.05–115)	126.86 (120.49–133)	<0.001
Glycerol (μmol/L)	323.3 (310.91–335)	254.26 (243.25–265)	634.2 (594.70–674)	<0.001
Ratio L/P	37.76 (34.91–40.6)	28.46 (27.15–29.7)	79.68 (65.51–93.8)	<0.001

The *p*-value refers to the difference between DCI and No-DCI hemispheres.

### Safety protocol

No complications directly related with the catheters were registered. Daily revisions of the catheter’s wounds were performed and no catheter infection was documented. Furthermore, none of the postoperative CT scans showed any catheter-related hemorrhage larger than 5cc.

## Discussion

Cerebral infarctions are likely to occur after aneurysmal SAH. These ischemic lesions are located in territories suffering angiographic vasospasms in 25–81% of cases^4,13,14^. The rest of brain infarctions cannot solely be explained by vasospasm, revealing the existence of other mechanisms of brain injury and raising the need of other diagnostic tools. CMD, as part of a multimodal neuromonitoring strategy, might improve delayed cerebral ischemia prediction. CMD offers direct metabolic information. However, this information is locally constrained and is not updated in real-time. Data from CMD probes is highly influenced by their location in the brain parenchyma and it might be misleading to extrapolate this information to interpret what is the real condition of the entire brain^15^. If SAH is considered a global cerebral disease, the location of the catheter would not be such a critical decision and one CMD probe might be enough to acquaint the metabolic profile of the brain as a whole. Nonetheless, as outlined by other authors, we believe that brain injury related to SAH may differ depending on the susceptibility of the different parenchymal or vascular territories^16,17^. Our results support this assumption proving the existence of infarctions in the silent or the DCI low-risk hemisphere thus providing rationale for considering bilateral CMD monitoring.

Subarachnoid blood volume in the initial CT after aneurysmal SAH has been defined as an independent prognostic factor^12^. Lagares *et al*. proved that cisternal blood volumes over 20 cc are clearly associated with an increased risk of poor outcome. In our series of 16 patients, the average volume of cisternal blood was 36 cc (SD: 21.7), supporting our intention to select severely affected patients. Despite these massive volumes, in 50% of our patients, interhemispheric difference in blood volume was lower than 5 cc. This condition reveals how difficult might be to initially select which hemisphere is more exposed to delayed injury. Strategies to determine the location of CMD catheters are based on the belief that the hemisphere containing the greatest volume of blood is the most susceptible to suffer deleterious changes. Tholance *et al*. developed an algorithm to determine the optimal location of CMD catheters in order to early detect DCI occurrence^18^. They improved the detection of DCI from 54% when the catheter was implanted in the hemisphere with more blood, to 79% when implementing this algorithm instead^18^. According to these authors, CMD catheters should be placed in the ACA territory for Anterior communicating artery (ACom) aneurysms and in the ipsilateral hemisphere for Middle cerebral artery (MCA), Internal carotid artery (ICA) and Posterior communicating artery (PCom) aneurysms. Nonetheless this algorithm failed to detect at least 20% of DCI. They also noted that territories where DCI was found had previously shown high LPR values. This finding supports the relevance of local measures in SAH. Our results concur with these statements, since CMD catheters from areas developing DCI showed clear differences compared to those located in areas which did not show any delayed brain injury.

In this clinical research, ischemic events were defined by validated values of LPR and Glucose^7,19^. Given the scarce therapeutic strategies to prevent DCI, we prioritized CMD to be specific, thus we used a restrictive definition of ischemia. Other scenarios deemed to increase the sensibility of CMD to predict DCI such as mitochondrial dysfunction (Increase in LPR, depending on Lactate with normal or increased pyruvate) or LPR augmentation without glucose decrease, were not considered^20^. Importantly, our LPR values were comparable to those detailed by other authors in critical patients^11,17^.

CMD has consistently proven its ability to display metabolic changes in different areas surrounding a focal concussion in the setting of traumatic brain injury (TBI)^21^. Therefore, a rationale exists for the use of multiple CMD catheters in patients suffering TBI^22^. However, this practice has not been established nor generalized in SAH. Jacobsen *et al*. recently reported their experience with multiple CMD catheters in SAH patients; nonetheless, only one hemisphere was monitored, and the detection of contralateral infarctions was not assessed^20^. Unlike TBI, diffuse and bilateral delayed infarctions in SAH are rare and probably the result of severe ischemic damage taking place within the very first hours. Hence, global DCI might be present in terminal SAH patients without any chance of therapeutic intervention^23^. Our results support the singularity of diffuse ischemia in SAH, since synchronic bilateral metabolic ischemic events hardly occurred for 5 hours. Therefore, if global infarction in SAH is that rare, the metabolic changes registered with one catheter should be extrapolated to the rest of the brain with extreme caution. The high efficiency of CMD measuring local changes in brain metabolism could be properly extrapolated if multiple catheters would be implemented^15^. In 5 out of 16 patients (31.25%) we observed cerebral infarctions, in one of them in a pre-considered silent territory. Herein CMD proved its high sensitivity predicting DCI, since every established infarction was preceded by various records of ischemic metabolism.

Our bilateral CMD monitoring strategy allowed us to detect that out of the total of ischemic events, 24% occurred in pre-considered silent hemispheres (b). These ischemic events came with few or no changes in the contralateral hemisphere (a). These findings match with the current evidence that defends that brain infarctions also occur in silent territories^6^. However, the majority of ischemic events do not lead to DCI, either because they do not last long enough or in response to the implementation of therapeutic measures that prevent infarction from being established. Nonetheless, some patients unavoidably develop permanent ischemic lesions despite of specific therapeutic attempts. Several studies have conferred CMD a high positive predictive value on DCI prediction^18^. In our series, CMD demonstrated a sensitivity and specificity of 96% and 83% respectively, when a threshold of 7 or more ischemic events was used. Despite the small size of our sample, these results are equal or superior to those reported by other authors^17,18^.

Summarizing, herein we have exposed a novel neuromonitoring strategy for SAH based on the bilateral use of CMD probes. This strategy allowed translating focal data into global metabolic trends in the brain. As stated in previous reports, CMD proved to be safe and useful. None of our patients suffered infections nor significative hemorrhages (<5 mm^3^) related to probe implantation. Furthermore, our results show that metabolic deficits detected by CMD rarely occur simultaneously and globally in SAH, affecting non-synchronously different brain territories. Nevertheless, risk-benefit balance should be carefully regarded since the rather small individual risk of implantation of multiple probes might occur, resulting in life threatening complications.

This study is not without limitations. It is important to note that this outstanding performance of CMD in our series might be due to a selection bias, since all the patients were severely affected by SAH (WFNS grades 4 and 5). Hence, it is expected that CMD predictive value would decrease in a more heterogeneous sample of patients. Another limitation of this study worth mentioning is the need to deepen the prediction of bilateral CMD monitoring in the case of posterior circulation aneurysms. A future cohort of patients with posterior fossa aneurysms may be necessary to determine the utility of these resources in these selected patients. Finally, despite obtaining sound statistically significant results, the limited number of patients calls for cautious interpretation. Our findings represent an intriguing starting point for further research but might not justify bilateral monitoring on their own.

## Conclusion

In selected SAH patients, bilateral CMD catheters seem to be safe and potentially useful to predict ischemic events that would have remained undiagnosed with a unilateral strategy. Almost a quarter of metabolic crisis patterns would have been silent if only one hemisphere had been monitored. Actually, less than 1% of ischemic events occurred simultaneously in both hemispheres. These findings suggest that careful extrapolation of data from unilateral monitoring should be considered. In the absence of effective focal therapies, adhering to locally constrained measurements may mislead our therapeutic decisions resulting in harmful consequences for the unmonitored brain.
